# Potential Impact of Geomagnetic Field in Transcranial Magnetic Stimulation for the Treatment of Neurodegenerative Diseases

**DOI:** 10.3389/fnhum.2017.00478

**Published:** 2017-09-27

**Authors:** Kwon-Seok Chae, Yong-Hwan Kim

**Affiliations:** ^1^Department of Biology Education, Kyungpook National University, Daegu, South Korea; ^2^Department of Nanoscience & Nanotechnology, Kyungpook National University, Daegu, South Korea; ^3^Brain Science and Engineering Institute, Kyungpook National University, Daegu, South Korea; ^4^Department of Biological Sciences, Neuroscience Program, Delaware State University, Dover, DE, United States

**Keywords:** transcranial magnetic stimulation, neurodegenerative diseases, geomagnetic field, electromagnetic fields, radical pair mechanism, imprinting

## Abstract

Throughout the long history of various therapeutic trials of transcranial magnetic stimulation (TMS), some TMS protocols have been reported to be clearly effective in the treatment of neurodegenerative diseases. Despite promising results from repetitive TMS (rTMS) using low frequency electromagnetic fields (EMFs) for neurodegenerative diseases, the low reproducibility has hampered the clinical applications of rTMS. Here, based on the notion of radical pair mechanism explaining magnetoreception in living organisms, we propose a new perspective that rTMS with controlled geomagnetic field (rTMS-GMF) can be an efficient and reproducible therapeutic approach for neurodegenerative diseases. In addition, combined consideration of imprinted GMF and/or EMFs in patients’ earlier life may augment the potential efficacy of the rTMS-GMF. The investigation of this approach is intriguing and may have a high impact on the technical suitability and clinical application of the rTMS-GMF in the near future.

## Background

Transcranial magnetic stimulation (TMS) using a variety of low frequency electromagnetic fields (EMFs) has been used in many ways for diagnosis and treatment of physical and psychological state or disorders such as cortical motor excitability, Parkinson’s disease (PD), Alzheimer’s disease (AD), epilepsy, stroke, pain, multiple sclerosis (MS) and depression (Boccard et al., 2015; Chervyakov et al., 2015; Kedzior et al., 2016; Kimiskidis, 2016; Lüdemann-Podubecká and Nowak, 2016). There are two categories of the EMFs used for repetitive TMS (rTMS) by frequency: low-frequency rTMS (≤1 Hz) and high-frequency rTMS (>1 Hz); the intensity of the EMFs used *in vitro* and *in vivo* ranges up to 10 tesla (Chervyakov et al., 2015). The effects of EMFs used in the rTMS trials are regarded as non-thermal and impossible to cleave covalent bonds in biomolecules due to the non-ionizing properties of the EMFs frequency (Chervyakov et al., 2015).

Throughout the long endeavor of various therapeutic trials of TMS, some TMS protocols have been reported to be clearly effective in the treatment of neurodegenerative diseases (Lefaucheur et al., 2014). For example, as Chervyakov et al. (2015) addressed, the clinical potential of rTMS for treating PD can be very high since many reports suggested that the magnetic stimulation can be beneficial for dopamine production including the up-regulation of tyrosine hydroxylase (TH) and NeuN, neuronal marker in the substantia nigra (Funamizu et al., 2005). Although the potential applications of TMS for neurological disorders have been highly valued, the reproducibility or reliability of rTMS application has been a big issue for clinical applications (Chervyakov et al., 2015). In line with this, a handful of reports suggested potential determining factors of the variability such as prior activity, attention, time of testing, age and gender (Sale et al., 2007; Ridding and Ziemann, 2010; López-Alonso et al., 2014; Vallence and Ridding, 2014; Vallence et al., 2014). In addition, recently Héroux et al. (2015) reported that insufficient sample size, questionable research practices and publication bias might be contributing factors for the low reproducibility (~50%) in the human motor cortical excitability.

In an intensive review of promising effects of rTMS, Chervyakov et al. (2015) suggested that TMS may be effective through non-classical biophysical interaction of magnetic fields such as the genetic magnetoreception, which is supported by the accumulated evidence through advancement of electromagnetic biology in the last two decades. So called the radical pair mechanism raises the notion that the lifespan of spin state for unpaired electrons in the flavin adenine dinucleotide (FAD) in cryptochrome, a putative magnetoreceptor protein, could be changed by geomagnetic field (GMF; 35–65 μT; Ritz et al., 2000; Hore and Mouritsen, 2016). In fact, cryptochrome is present in virtually all living organisms such as plants and animals including human beings and expressed in most organs and tissues including brain (Lin and Todo, 2005). At the initial step of the GMF sensing by cryptochrome, the intensity of GMF and its inclination that is the angle measured from the horizontal plane to the GMF vector, are considered to play critical roles in inducing GMF effects (Ritz et al., 2000; Hore and Mouritsen, 2016). The functional activation or inactivation of cryptochrome is exerted by conformational changes at the active site harboring the FAD, which in turn modulate the interaction between cryptochrome and adjacent biomolecules in signaling pathways, and eventually manifest a plethora of multifaceted biological events such as magnetoreceptive migration, geotactic behaviors, modulation of circadian rhythm (Partch and Sancar, 2005; Yoshii et al., 2009; Fedele et al., 2014; Bae et al., 2016; Hore and Mouritsen, 2016). Interestingly, several recent studies clearly demonstrated that GMF can be sensed by a set of neurons in animals and human brains. In nematode (*C. elegans*), the local GMF (48 μT) was directly sensed by a pair of thermosensory neurons called AFD (amphid neurons with finger-like) in the head that required intact TAX-4 cGMP-gated ion channel for magnetic orientation and vertical burrowing migration (Vidal-Gadea et al., 2015). In fruit flies, the GMF (50 μT) appeared to be sensed by neurons of the Johnston’s organ in the second segment of the antennae to modulate geotactic upward and downward behaviors (Bae et al., 2016). In addition, the light sensitivity of the human visual system in identifying a light dot with different brightness on the screen was significantly dependent upon the modulated direction of the GMF (48 μT; Thoss et al., 2000, 2002). Moreover, the fluctuated GMF by the solar storms (disturbance range: 21–500 nT) or the simulated GMF by the solar storm (7 Hz; 0, 20 and 70 nT peak intensities) induced significantly altered electroencephalograms in a couple of brain regions including prefrontal and right parietal cortex, indicating the occurrence of magnetosensory evoked potentials and accompanied emotional changes in some cases (Babayev and Allahverdiyeva, 2007; Mulligan et al., 2010; Mulligan and Persinger, 2012). The results underscore that GMF with a negligible rate of change can influence neuronal functions. In particular, functional existence of cryptochrome in the neurons was necessary for the GMF-induced magnetosensitive neuronal activations in the fly studies (Vidal-Gadea et al., 2015; Bae et al., 2016), supporting cryptochrome as the magnetoreceptor in the neuronal cells. Together, these studies suggest that GMF affects neuronal function and possibly modulate TMS effects on the brain.

## Suggestive Approach

**Figure 1:**
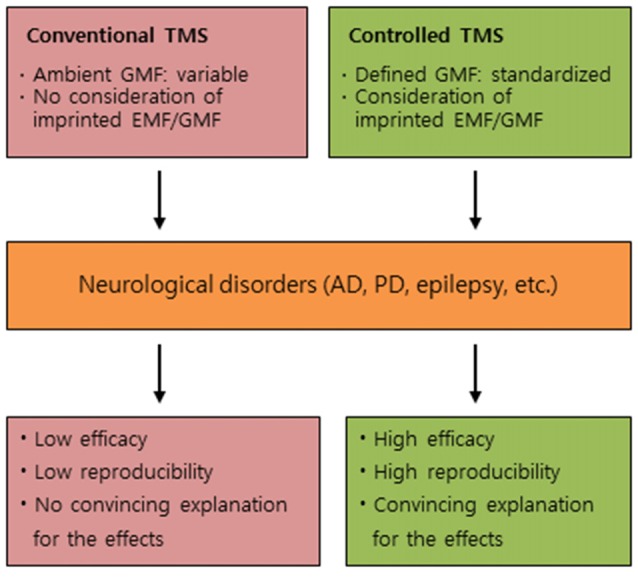
Conceptual differences between conventional TMS and controlled TMS. A comparative summary for the effects of conventional TMS and controlled TMS on neurological disorders. TMS, transcranial magnetic stimulation; GMF, geomagnetic field; EMF, electromagnetic field; AD, Alzheimer’s disease; PD, Parkinson’s disease.

We would like to address other perspectives, in addition to listed factors based on anonymous surveys from researchers in the TMS, revealing the prevalence of non-reproducible results in TMS research and questionable research practices in the field (Héroux et al., 2015). We propose to consider potential roles of GMF and magnetic imprinting as new perspectives in the underlying mechanism of TMS effects, and suggest that the controlled GMF/EMFs in TMS can be an efficient therapeutic approach to induce high reproducibility in the clinical trials of neurological disorders, as depicted in Figure 1.

The GMF penetrates most materials, not only natural entities including woods, hills and mountains, but also man-made objects such as houses, buildings and instruments. However, because of the differential permeability of materials and the complex grids of various EMFs in the surroundings, natural GMF, i.e., the parameters of GMF are distorted, nonlinear and contaminated with artifacts virtually everywhere, especially in artificial places, e.g., hospital, research and business buildings, and private houses (Engels et al., 2014). Such various weak EMFs’ contamination could disrupt a radical pair mechanism-based magnetoreceptive orientation of living organisms including birds. Considering the basic notion of the radical pair mechanism above, we would like to emphasize that the local anomaly of GMF such as its intensity and inclination or EMFs might be a critical factor for inducing the low reproducibility of TMS effects. Therefore, if that is the case, it is desirable to define ambient GMF to provide patients with “controlled” TMS treatment. First, it is required to decide whether to keep GMF constant (constant GMF) during the time of treatment or to keep it variable by the GMF in nature (natural GMF), since GMF fluctuates by the time of day, season and year (Finlay et al., 2010). In addition, stray EMFs are needed to be excluded from the treatment room using passive and/or active shielding gear in both cases above (Engels et al., 2014). This may prevent potential artifacts and unintended effects from EMFs surrounding the treatment room. To the best of our knowledge, there has been no study to investigate the effect of TMS under a controlled GMF or EMF. It is important to assess which is more effective in the TMS treatment, constant GMF or natural GMF, for enhancing the clinical reproducibility and efficacy.

Second, EMF/GMF imprinting may be a contributing factor for effective TMS treatment. This notion derives from the possibility that imprinted EMF or GMF information affects TMS effects. There is some experimental evidence supporting the concept that the sensitivity to EMF in human health may be influenced by previous exposure to power-line frequencies (Blackman, 2006). In hatched chicks, Ca^2+^ efflux in the hippocampus was elicited by 50 or 60 Hz magnetic fields. Intriguingly, the magnetic field-induced efflux was significantly dependent on the frequency of the magnetic field (50 or 60 Hz) that was applied to the developing eggs. In the case of GMF, more convincing evidence has been accumulated. Initially, the GMF imprinting hypothesis was suggested to explain how loggerhead sea turtles navigate the home-coming to the Florida seashore across the Atlantic Ocean (Lohmann et al., 2008). This hypothesis has been supported by several studies, providing empirical evidence that imprinted GMF information at particular hatching places appears to be stored and used to navigate long distances (~hundreds to thousands km) by sea turtles and salmons (Putman et al., 2013; Brothers and Lohmann, 2015). Although the scope of imprinting can be varied, EMF/GMF imprinting in earlier human life could be possible on babies and chicks, based on recent comparative studies. Newborn babies and naïve chicks showed similar susceptibility for looking preference to animate cues such as semi-rigid motion for walking chicken, which were provided by point light-displays on the screen (Simion et al., 2008; Di Giorgio et al., 2017a) and association with self-propelled objects over passively moving objects caused by physical contact (Mascalzoni et al., 2010; Di Giorgio et al., 2017b). The potential susceptibility may be derived from either an innate predisposition or imprinted behaviors that were inherited at least in part from ancestors including parents. In addition, various epigenetic factors including stress, fetal hypoxia and hypertension during gestation can modulate fetal brain shaping and influence later development of neurodegenerative diseases such as AD and PD (Faa et al., 2014). Therefore, pre-exposed EMF/GMF to the parents or fetus could be imprinted in the brain and/or other organs of a newborn baby and it may affect the efficacy of TMS on neurodegenerative diseases afterward.

**Figure 2:**
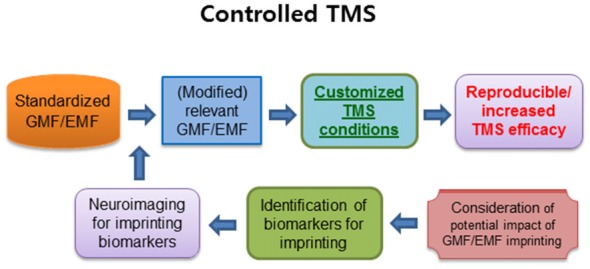
The summary of a critical approach for reproducible and increased TMS efficacy. The controlled standardized GMF/EMF may induce higher reproducibility as well as an increase in TMS efficacy. In addition, the pre-exposed EMF/GMF may generate magnetic imprinting, which can be another factor for customizing the GMF/EMF conditions. The identification of imprinting biomarkers for neuroimaging may have a profound impact on customized TMS therapy to enhance the TMS efficacy. TMS, transcranial magnetic stimulation; GMF, geomagnetic field; EMF, electromagnetic field. Arrows indicate anticipated outcomes.

If this is the case, it is important to characterize the possibly imprinted information, in terms of the location and scale as well as identifying region-specific potential biomarkers (Figure 2). Recent studies showed that 50 Hz power frequency magnetic fields produced remarkably altered methylation profile of genomic DNA in human neural cells (Giorgi et al., 2017) and micro RNAs-mediated deregulation of important signaling pathways in mouse spermatocyte-derived cells (Liu et al., 2015). Moreover, the low intensity (7 mT) of static magnetic fields increased DNA methylation and polymorphism in the callus of wheat embryos (Aydin et al., 2016). The results in developing cells suggest that alterations in the expression of micro RNAs, methylation and polymorphism in DNA, and cell signaling pathways, could be potential biomarkers for the assessment of EMF/GMF imprinting. Although the potential link has been barely studied to date, it is noteworthy to assess the potential impact of EMF/GMF imprinting on TMS treatment and its modified application. For example, in case of EMF imprinting, it may be possible to compare TMS effects between two groups of patients; a group of patients exposed to high dose of power frequency magnetic fields in their infancy vs. a group with normal dose of the magnetic fields in earlier life. For GMF imprinting, we can compare TMS effects on the groups of e.g., PD patients who lived their infancy period in either higher or lower latitude places. Since the prevalence of MS can be related to the temperature climate, geographic location and possibly GMF (Ascherio and Munger, 2007; Milo and Kahana, 2010), it is interesting to assess the potential implication of GMF imprinting in TMS treatment. When the potential impact of EMF/GMF imprinting on TMS treatment is characterized, appropriate modification of standardized GMF and/or EMF for TMS treatment will be considered for enhancing TMS efficacy in the patients. Furthermore, it could be very useful to identify biomarkers in displaying GMF/EMF imprinting using biomedical imaging devices, which help to establish customized TMS conditions for improving diagnostic or therapeutic applications, although there are no databases for the approach yet (Figure 2).

## Conclusion

Numerous positive effects of TMS on humans raised scientific and clinical interests. However, due to the low reproducibility and lack of convincing mechanistic understanding of TMS effects, diagnostic or therapeutic applications have been seriously hampered. Once the proposed idea is confirmed to be plausible, clinical TMS trials can be conducted under relevant conditions. Then more reproducibly promising outcomes would be broadly expected, which is summarized in Figure 2. Furthermore, if the TMS effect on the diseases is dependent on the imprinted information of EMFs and/or GMF based on the critical window of earlier life, we may even consider imprinting one’s brain or whole body with a particular GMF and/or EMFs in advance, to elevate the effects of TMS in future. In a promising perspective, accumulated data of GMF/EMFs dosimetry and clinical outcome of TMS could be exploited as a foundation for a personalized TMS diagnosis or therapy for neurological states or disorders.

## Author Contributions

K-SC wrote the article and is responsible for handling the manuscript. Y-HK provided critical revisions and final approval and have an equal responsibility for the article.

## Conflict of Interest Statement

The authors declare that the research was conducted in the absence of any commercial or financial relationships that could be construed as a potential conflict of interest.
